# T-Cell Exhaustion in Chronic Infections: Reversing the State of Exhaustion and Reinvigorating Optimal Protective Immune Responses

**DOI:** 10.3389/fimmu.2018.02569

**Published:** 2018-11-09

**Authors:** Alireza Saeidi, Keivan Zandi, Yi Ying Cheok, Hamidreza Saeidi, Won Fen Wong, Chalystha Yie Qin Lee, Heng Choon Cheong, Yean Kong Yong, Marie Larsson, Esaki Muthu Shankar

**Affiliations:** ^1^Department of Medical Microbiology, Faculty of Medicine, University of Malaya, Kuala Lumpur, Malaysia; ^2^Center of Excellence for Research in AIDS, University of Malaya, Kuala Lumpur, Malaysia; ^3^Department of Pediatrics School of Medicine Emory University, Atlanta, GA, United States; ^4^Department of Biomedical Sciences, Faculty of Medicine and Health Sciences, University of Putra Malaysia, Selangor, Malaysia; ^5^Laboratory Center, Xiamen University Malaysia, Sepang, Malaysia; ^6^Division of Molecular Virology, Department of Clinical and Experimental Medicine, Linköping University, Linköping, Sweden; ^7^Division of Infection Biology and Medical Microbiology, Department of Life Sciences, School of Life Sciences, Central University of Tamil Nadu, Thiruvarur, India

**Keywords:** T-cell exhaustion, PD-1, immunotherapy, rejuvenation, T-bet, metabolism, epigenetics

## Abstract

T-cell exhaustion is a phenomenon of dysfunction or physical elimination of antigen-specific T cells reported in human immunodeficiency virus (HIV), hepatitis B virus (HBV), and hepatitis C virus (HCV) infections as well as cancer. Exhaustion appears to be often restricted to CD8+ T cells responses in the literature, although CD4+ T cells have also been reported to be functionally exhausted in certain chronic infections. Although our understanding of the molecular mechanisms associated with the transcriptional regulation of T-cell exhaustion is advancing, it is imperative to also explore the central mechanisms that control the altered expression patterns. Targeting metabolic dysfunctions with mitochondrion-targeted antioxidants are also expected to improve the antiviral functions of exhausted virus-specific CD8+ T cells. In addition, it is crucial to consider the contributions of mitochondrial biogenesis on T-cell exhaustion and how mitochondrial metabolism of T cells could be targeted whilst treating chronic viral infections. Here, we review the current understanding of cardinal features of T-cell exhaustion in chronic infections, and have attempted to focus on recent discoveries, potential strategies to reverse exhaustion and reinvigorate optimal protective immune responses in the host.

## Introduction

T cells play a key role in orchestrating pathogen-specific adaptive immune responses. During primary infection, naive T cells recognize antigenic peptides presented on major histocompatibility complex (MHC) molecules via their T-cell receptors (TCRs), leading to their activation and differentiation into effector T cells over the course of ~1–2 weeks (1, 2). This differentiation results in robust proliferation, transcriptional, epigenetic, and metabolic changes, as well as the acquisition of effector functions, altered tissue homing, and massive clonal expansion (1, 3). Following antigenic clearance, a vast majority of the effector T cells die by apoptosis. However, ~5–10% of the cells persist and differentiate into memory T cells such as central memory, effector memory, and tissue resident memory T cells (1, 3). Memory T cells are maintained after the effector phase and can rapidly execute their effector functions in response to reinfection/exposure to previously encountered antigens. The rapid effector function arises when the antigen is present transitory during an acute infection. Nonetheless, this programming of memory T cell differentiation is distinctly altered during chronic viral and bacterial infections, and also in chronic diseases such as cancer due to persistent antigenic exposure and/or inflammation (3, 4). When altered differentiation progresses, the immune response fails, and antigen-specific T cells progress to a state called T-cell exhaustion.

T-cell exhaustion was first defined in 1993 by Moskophidis and colleagues when they demonstrated impaired cytotoxic functions during viral persistence in murine models (5). T-cell exhaustion denotes the physical elimination of antigen-specific T cells, also observed in chronic lymphocytic choriomeningitis virus (LCMV) infection of mice (6, 7). T-cell exhaustion has also been reported in various human chronic viral infections such as human immunodeficiency virus (HIV), hepatitis B (HBV), and hepatitis C (HCV), as well as in cancer (4, 8, 9). Exhaustion has been mostly described for CD8+ T cells responses although CD4+ T cells have also been reported to be functionally unresponsive in several chronic infections (10, 11). Wherry et al. firstly described the molecular signature of CD8+ T-cell exhaustion during chronic viral infection (12). Later, advances in biomedical technologies in research including the utilization of MHC multimers that can recognize antigen-specific T cells without reliance on T cell functions as readout, as well as the progress in improved methods to evaluate the phenotypic and functional portfolios of single cells have enhanced our understanding of the complexities underlying the T-cell exhaustion phenomenon (10, 13).

The state of exhaustion is mainly characterized by sequential loss of T cell effector functions in a hierarchical manner where loss of interleukin (IL)-2 production is the earliest sign (14, 15). Subsequently, the production of tumor necrosis factor (TNF) and other cytokines is dramatically reduced. However, interferon (IFN)-γ, beta-chemokine production and perhaps cytotoxic activities, are more resilient to inactivation (16–19). Exhausted T cells also have altered proliferative abilities, sustained upregulation of a wide array of co-inhibitory receptors, unique transcriptional and epigenetic signatures, altered metabolic fitness, failure for transition to quiescence, and acquisition of antigen-independent memory T cell responsiveness (3, 4, 8, 20, 21). Notably, severely exhausted T cells appear to undergo apoptosis and become eliminated leading to marked decline in virus-specific T cells (5, 22, 23).

It is imperative to also understand that T-cell exhaustion should be clearly delineated from T-cell anergy and senescence. T-cell anergy is a state of non-responsiveness molecularly distinct from T-cell exhaustion, which is induced by excessive stimulation of TCR and either robust co-inhibitory molecule signaling or restricted presence of concomitant co-stimulation through CD28 (4, 24). On the other hand, senescent T cells are often described by increased expression of markers such as killer-cell lectin-like receptor G1 (KLRG1) and/or CD57 (25–27), which exhausted T cells have in low levels on their surfaces (12). The expression of PD-1 is also increased on exhausted T cells whereas senescent cells seldom express this marker (28). In this review, we will discuss our current understanding of cardinal features of T-cell exhaustion in chronic infections, while we will attempt to also focus on recent discoveries and potential strategies for reversing the state of exhaustion with a view to reinvigorate immune responses.

## Immune checkpoints with therapeutic potentials in T-cell exhaustion

In acute infections, co-inhibitory receptors function to dampen the magnitude of immune responses, which are in fact, down-regulated after pathogen clearance to achieve homeostasis, and development of memory T cells. However, this pattern diverges during chronic infections, where higher and sustained expression of co-inhibitory receptors is characteristic (3, 10). Co-inhibitory receptors vary in expression pattern, ligands, and signaling motifs and our understanding of the molecular mechanisms whereby they control T-cell exhaustion is seldom understood (29). However, the identification of their significance in the dysregulation of cellular immune responses in chronically infected hosts has provided newer avenues for designing therapeutic molecules to restore optimum immune responses (10).

## PD-1 plays a pivotal role in regulating T-cell exhaustion

The dominant role of programmed death-1 (PD-1) in regulating T-cell exhaustion was first revealed following gene expression profiling of virus-specific CD8+ T cells during chronic LCMV infection (30). Exhausted T cells up-regulated PD-1 expression, and blockade of the PD-1 pathway promoted effector functions of virus-specific T cells and significantly reduced the viral load in the experimental animals. This result has been further substantiated in many other chronic infections in mice, non-human primates, and humans.

During HIV-1 infection, PD-1 expression on HIV-specific CD8+ T cells positively correlates with high viral load, impairment of CD8+ T-cell function, disease progression, and reduced CD4+ T-cell counts. Antiretroviral therapy (ART) can reduce the expression of PD-1 on virus-specific T cells in HIV-infected patients. Long-term non-progressors (LTNPs) have low expression of PD-1 on virus-specific T cells and these populations are more polyfunctional than T cells of progressors (10, 31, 32). *In vitro* studies describe that blocking the PD-1 pathway restores T-cell functions and improves pathogen control by enhancing the proliferation potentials of T cells and promoting cytokine production (31–33). Moreover, *in vivo* administration of anti-PD-L1 antibody increased both CD4+ and CD8+ T cells with the ability to inhibit viral replication, i.e., decreasing the plasma viral load, in mice chronically infected with HIV-1 (34). More recently, treatment with PD-1 inhibitory antibody during simian immunodeficiency virus (SIV) infection increased the frequencies and functional quality of SIV-specific CD8+ T cells detectable in the blood and gut, viral loads declined, and significantly improved the survival rates in infected macaques (35, 36). In addition to HIV, the dynamics and significance of the PD-1 pathway has been investigated in HBV and HCV infections (37–41). In chimpanzees chronically infected with HCV, a 100-fold suppression of viremia was observed in one of three animals treated with anti-PD-1 antibodies. Control of virus replication was associated with reinvigoration of HCV-specific CD4+ and CD8+ T cell responses (42). Interestingly, PD-1 expression noticeably increased on HCV-specific CD8+ T cells in the liver although the blocking of PD-1 had no enhancing effect on the functions of these cells (41). This explains that multiple factors must contribute and control the maintenance of T-cell exhaustion and also indicates that the severity of exhaustion is highly influenced by the location and levels of viral antigen and the compartmentalization of the virus-specific T cells (10).

Clinical trials have so far only evaluated single-dose regimens in chronically infected patients, due to considerations of potential toxicities of PD-1-targeted therapy in otherwise healthy individuals (29). Even though there was only a modest response rate for chronic HCV, among 20 patients receiving the highest anti-PD-1 dose, three showed remarkable reduction in viral RNA, and in 1 patient, HCV was undetectable for at least 1 year. Mild to moderate immune-related adverse events were reported in six of 54 patients, which were resolved without specific intervention (43). Single-dose PD-1-targeted therapy, i.e., anti-PD-L1, has been evaluated in HIV infected patients on clinically effective combination ART (cART). In this study, Gay et al. described an increase in HIV-specific CD8+ T cell responses in the blood in two of six patients, but without any effects on HIV viral load. This result could likely be attributed to the dosage of anti-PD-L1 antibodies used, which was 10-fold lower than dosages selected for activity in patients with cancer (44). These clinical trials suggest that there is potential to use PD-1-targeted therapy in some patients for overcoming chronic infections and that combination treatments should further be assessed (29).

## Contribution of other co-inhibitory receptors for T-cell exhaustion

There are several co-inhibitory molecules other than PD-1, which are expressed on exhausted T cells. Exhausted T cells can co-express PD-1 together with cytotoxic T lymphocyte antigen-4 (CTLA-4), T cell immunoglobulin domain and mucin domain-containing protein 3 (TIM-3), 2B4 (CD244), lymphocyte activation gene 3 protein (LAG-3), CD160, and several others (45). The individual expression of PD-1 or other co-inhibitory receptors does not define a state of exhaustion rather a co-expression of multiple co-inhibitory receptors do. Interestingly, the indicated co-expression patterns are mechanistically related, as concurrent blockade of these multiple co-inhibitory receptors lead to synergistic reversal of exhaustion (3). Direct *in vivo* blockade of CTLA-4 during chronic viral infections such as LCMV, SIV, and HIV suggest that blockade of CTLA-4 fail to decrease the viral load or increase T cell functionalities (30, 46). In HCV infection, *in vitro* blockade of PD-1 alone failed to restore the functions of hepatic PD-1+ CTLA-4+ virus-specific CD8+ T cells although concurrent blockade of CTLA-4 and PD-1 reinvigorated HCV-specific CD8+ T cells in a CD4+ T cell–independent manner (41). Impressive results in controlling cancer has been demonstrated for combined PD-1 and CTLA-4 blockade in patients with melanoma, and drugs targeting at least three other immune checkpoints, i.e., LAG-3, TIM-3, and TIGIT, are now in clinical trials (29, 47). The investigation of co-inhibitory molecules in co-regulating T-cell exhaustion indicates that these pathways are non-redundant. It is also important to consider that the molecules constitute diverse structural families, which bind ligands with unique expression patterns and poses distinct intracellular signaling pathways. Hence, there is a potential to negate exhaustion through manipulation of the pathways where these molecules are involved (3).

## Cytokines and T-cell exhaustion

Cytokines are molecules that facilitate communications, activation, differentiation and de-activation of immune cells. Involvement of both pro- and anti-inflammatory cytokines in T-cell functions has been extensively studied to understand the physiology of T cells under stressed conditions (48). Of these modulators is the immunosuppressive cytokine IL-10, which has been identified as a potential target to reinvigorate exhausted T cells. IL-10 has been reported to be positively associated with persistence of viral infections such as HCV, HIV, and Epstein-Barr virus (EBV), a possible strategy for viruses to evade host immune defenses (49, 50). In LCMV infection models, the blockade of IL-10 appears to inhibit viral persistence and enhances T-cell functions (51, 52). IL-10 blockade is also employed in HIV infection, whereby IL-10Rα blockade results in markedly increased secretion of IFN-γ by CD4+ T cells. However, combining IL-10 blockade with PD-1 blockade can only restore a restricted number of cytokines produced by T cells including IL-2 (53). On the contrary, in a mouse model of LCMV infection, combined anti-IL-10 and anti-PD-1 therapy synergistically enhanced anti-viral response of T cells (54). Despite the benefits of anti-IL-10 therapy, there are still disadvantages in modulating this otherwise important anti-inflammatory cytokine. For instance, IL-10 production and downstream signaling paves the way for regulating inflammation against gut microbiome (55). Taken together, with the observations of IL-10-mediated regulation of liver inflammation following LCMV infection, treatment with anti-IL-10 should be carefully calibrated to prevent undesirable side-effects (56). This concern also warrants a thorough revisit on the role of IL-10 blockade in immunotherapy (50).

Apart from IL-10, IL-2 also serves as a target for immunotherapy. Exogenous administration of IL-2 *in vitro* is able to abrogate PD-1 inhibitory signaling (57). Grint et al. demonstrated that IL-2 treatment successfully decreased the virus RNA levels in HCV in HCV/HIV co-infected patients (58). Importantly, IL-2 therapy is highly dependent on the state of infection and can give rise to opposing consequences, such as that previously described with the administration of IL-2/anti-IL-2 immune complexes, which results in expansion of regulatory T cells (Tregs) that impede virus clearance and anti-viral functions of T-cells (59).

Other cytokines serving as potential immunotherapeutic targets in reverting T-cell exhaustion include IL-7, IL-17, IL-21, IL-22, and many others, which are also regarded as key mediators during chronic infections (60–63). IL-21, for instance, improves the clinical outcome in SIV-infected macaques with reduction in the non-AIDS-associated morbidities when administered as supplements with probiotics and ART (64). Other combinations of ART with type-1 interferon (IFN-I) has also yielded positive results by reducing the viral loads and restoring CD8+ T-cell functions in humanized mice infected with HIV (65).

## Cross-talk of exhausted T cells with other immune cells

The frequency of Tregs may be increased in HIV and HCV infections where Tregs likely limit the *in vitro* responses of effector T cells (66). The direct role of Tregs in exhaustion of CD8+ T cells as well as FOXP3–CD4+ T cells remains unclear. There is also a possibility for Tregs to play a role in exhaustion considering that Tregs are a source of IL-10, TGF-β and perhaps other suppressive cytokines, for instance IL-35 (66). Interestingly, recent reports in a chronic LCMV model described an interaction between Tregs and the PD-1 signaling pathway in regulating exhausted CD8+ T cells because simultaneous reduction of Tregs and blockade of PD-1 signaling pathway appears to have a robust synergistic effect on viral control and reversal of exhaustion (67). Furthermore, other immune cell types such as antigen-presenting cells (APCs), myeloid-derived suppressor cells (MDSCs) (68, 69) natural killer (NK) cells and even CD8+ regulatory populations (70–72) have been reported to directly or indirectly promote T-cell exhaustion during chronic infections.

Accumulating evidence indicates that functional impairment of DCs could be associated with exhaustion of T cell functions and progression of disease in HIV, HBV, HCV, and LCMV infections (70, 71, 73–75) although the molecular mechanism behind the impairment of T cell functions mediated via DCs during chronic infections still remain ambiguous. Recent studies showed that DCs promote T-cell exhaustion through signaling by inhibitory receptors such as PD-1 and CTLA-4 (75, 76). Indeed, PD-L1 is up-regulated on mDCs, although MHC molecules and co-stimulatory molecules such as B7-1, B7-2, and CD40 are down-regulated (74, 75, 77). Intriguingly, up-regulated PD-L1 appears to impair DC functions and associate with disease progression during HIV and HBV infections (74, 75, 77).

## Transcriptomic changes in T-cell exhaustion

Several recent studies demonstrated that exhausted T cells have a transcriptional profile remarkably different from their effector and memory counterparts. These differences include major alternations in the expression level of co-inhibitory and co-stimulatory molecules, transcription factors, signaling molecules, chemokine and cytokine receptors, and genes involved in metabolism (12, 20, 78). There are several transcription factors that play significance roles in T-cell exhaustion, including T-bet, EOMES, FOXO1, FOXP1, Blimp-1, NFAT, BATF, IRF4, and von Hippel–Lindau disease tumor suppressor (VHL) (16, 79–86). Intriguingly, the main transcription factors, which regulate establishment of T-cell exhaustion function in a specific manner. During acute infection, terminally differentiated CD8+ T cells express T-bet that has a functional role in the development of these cell subsets (87) and differentiation of Th1 cells. However, this transcription factor regulates the population of non-terminal progenitor cells within the exhausted T cell subsets during chronic infections (2, 81). It is described that EOMES favor the development of central memory T cells during acute infection by regulating quiescence and homeostatic turnover (88–90). On the other hand, it is reported that EOMES regulates the development of a terminally-differentiated subset of exhausted T cells during chronic infection (81). There are two phenotypically characterized subsets of exhausted T cells that are described by intermediate expression of inhibitory receptor PD-1 and high expression of the transcription factor T-bet and (PD-1^Int^T-bet^Hi^) or high Eomes and high PD-1 expression (Eomes^Hi^PD-1^Hi^). Although both the populations are required for control of chronic infection, the PD-1^Int^ subset have been shown to contribute distinctly to clearance of pathogens upon PD-1 blockade (3, 81, 91).

In a study using toxoplasma encephalitis (TE)–susceptible model, the CD4 +T cells experienced a more pronounced exhaustion in comparison with CD8+ T cells. It has also been demonstrated that deletion of Blimp-1 from exhausted CD4+ T cells led to reversal of CD8+ T-cell exhaustion and improved pathogen control (92). The transcription factor interferon regulatory factor 9 (IRF9) has an integral role in the antiviral immune response and is considered as a component of IFN type I signaling pathway downstream of the IFN-I receptor (IFNAR) (93). Using LCMV acute infection model, it has been demonstrated that IRF9 limited early LCMV replication by controlling the expression of IFN-stimulated genes and IFN-I, and by regulating the levels of IRF7, a transcription factor necessary for IFN-I production. The study has also revealed that infection of IRF9- or IFNAR-deficient mice resulted in the loss of early restriction of viral replication and impaired anti-viral responses among dendritic cells, leading to CD8+ T-cell exhaustion and chronic infection (94). Our understanding of the transcriptional regulation of T-cell exhaustion is progressing, although it is important to elucidate the mechanisms controlling the altered pattern of gene expression. Moreover, there is also a necessity to identify a master lineage-specific transcription factor for exhausted T cells (63).

## Signaling pathways and T-cell exhaustion

**Figure 1:**
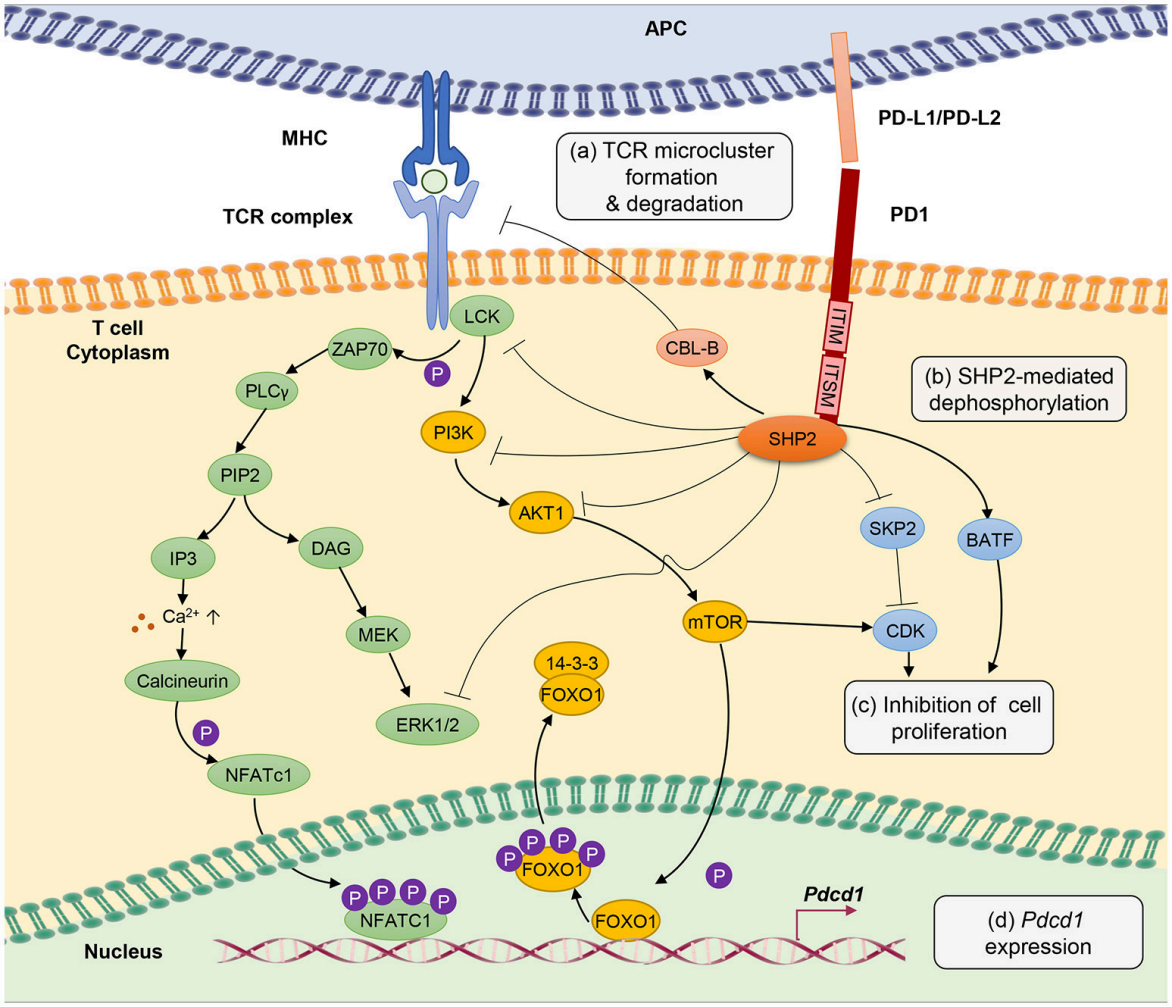
PD-1 signal inhibits T-cell receptor (TCR) signaling pathway through several different mechanisms. **(a)** PD-1 engagement with PD-L1 or PD-L2 ligands blocks TCR signal transmission by promoting microcluster formation and degradation of TCR. Accumulation of PD-1 within the synapse stabilizes the interaction between T cells and antigen presenting cells causing “immune paralysis” and cell motility arrest. PD-1 ligation also enhances TCR internalization and degradation by Casitas B-lymphoma (CBL)-B E3 ubiquitin ligase. **(b)** PD-1 signaling recruits SHP2 phosphatase to immunoreceptor tyrosine-based inhibitory motif (ITIM) and immunoreceptor tyrosine-based switch motif (ITSM). SHP2 dephosphorylates ZAP70, ERK1/2 and suppresses the phosphatidylinositide 3 kinase (PI3K)/AKT/Mammalian Target of Rapamyc from (mTOR) pathways, thus inhibiting multiple T-cell activation pathways. **(c)** PD-1 suppresses T-cell proliferation by blocking the transcription of Skp1/Cullin/F-box protein ubiquitin ligase (SKP2), which controls cyclin-dependent kinases (Cdks) activation. A Basic leucine transcription factor, ATF-like (BATF) is also a downstream target of PD-1 signaling that causes repression of T-cell proliferation and cytokine secretion. Besides, by inhibiting TCR signaling, PD-1 blocks IL-2 production to limit T-cell proliferation. **(d)** PD-1 signaling promotes FOXO1 retention in nucleus and enables *Pdcd1* gene transactivation. Nuclear factor of activated T cells (NFATc1) in the absence of AP-1 interaction promotes the expression of *Pdcd1* (PD-1 encoding gene). The transcription of *Pdcd1* is inhibited by AKT/mTOR signaling, which promotes phosphorylation of FOXO1 and 14-3-3 docking to sequester FOXO1 molecule from the nucleus into the cytoplasm. PD-1 signaling stops the process by targeting the AKT/mTOR pathway.

T-cell exhaustion involves a tight control by intricate signaling pathways, either through active or passive suppression (Figure 1). The signaling pathways involved in the up- and down-streams of PD-1, a central player in rendering T-cell exhaustion, are as discussed herein.

Chronic antigen stimulation alone is known to be adequate in conferring T-cell exhaustion and inducing PD-1 expression (3). PD-1 limits T-cell activation by attenuating TCR signaling, thus preventing immunopathology. PD-1 is a transmembrane molecule, and it's C-terminal domain at the cytoplasmic tail harbors an V/IxY225xxL/V immunoreceptor tyrosine-based inhibitory motif (ITIM) and an TxY248xxV/I immunoreceptor tyrosine-based switch motif (ITSM) inhibitory domains, which serve as binding sites for Src homology 2 domain-containing tyrosine phosphatase 2 (SHP2) (95–97).

Mutation of the ITSM tyrosine domain, which prevents SHP2 recruitment attenuates the ability of PD-1 to suppress T cell activation (96, 98). Engagement of PD-1 receptor with PD-L1 or PD-L2 recruits SHP2 phosphatase to its cytoplasmic domain, which functions to inhibit TCR signaling pathway by preventing ZAP70 phosphorylation and its association with CD3ζ at TCR complex (99). SHP2 also interferes with CD28 costimulatory signaling by blocking PKC-θ activation and dephosphorylates TCR signaling molecules including phospholipase C γ 1 (PLCγ1) and Extracellular signal-regulated protein kinases (ERK1/2) (100).

Besides, PD-1 antagonizes T-cell signaling by inhibiting phosphatidylinositide 3-kinase (PI3K)/AKT/mammalian target of rapamycin (mTOR) pathway. The PI3K/AKT/mTOR pathway is well established in regulating cell survival, proliferation and metabolism. Recruitment of SHP2 to the ITIM and ITSM cytoplasmic domains of PD-1 inhibits PI3K thus further blocks AKT phosphorylation and mTOR pathway (101). It is important to note that reports from cancer studies suggest that PD-1 engagement with PD-L1 does not cause dephosphorylation, but leads to phosphorylation and activation of AKT and ERK, which increases chemo resistance of the cancer cells (102, 103), implying a difference in PD-1 signaling between chronic infection and cancer that requires further investigation. Through inhibiting these signaling molecules, PD-1 indirectly inhibits the production of cytokines including IL-2, hence interferes with T cell growth and function. Anti-PD-1 treatment is able to restore anti-viral T-cell signaling such as phosphor-JNK, phosphor-ZAP-70 and phosphor-ERK, besides cytokine production (104).

T-cell activation promotes clathrin-independent internalization TCRζ internalization as reported by Compeer et al. (105) but TCR unit is often directed to the recycling compartment. PD-1 ligation antagonizes TCR signaling by causing internalization and ubiquitin-mediated degradation by casitas B-lymphoma (Cbl)-b E3 ubiquitin ligase (106). Besides, results from single cell imaging suggest that engagement of PD-1 receptors form a microcluster consisting of PD-1 and TCR within the synapse (100). This PD-1-TCR microcluster formation interferes with the TCR signal and mediates T-cell suppression. In case of chronic infection, viral persistence causes the arrest of T cell motility in the splenic red pulp, as PD-1 engagement provides stability to the immunological synapse resulting in immune paralysis in which the unresponsive T cell cannot be released from the sites to engage other targets (104). However, T-cell motility arrest can be completely restored by therapeutic blockade of intravenous injection of antibodies against PD-1 and PD-L1 (104).

One of the hallmarks of T-cell exhaustion is impaired cell proliferation. PD-1 inhibits cell proliferation by attenuating TCR-mediated activation of IL-2 production (99). In addition, PD-1 blocks cell cycle progression through the G1 phase by suppressing multiple transcription of SKP2, downstream of PI3K-AKT and ERK pathways (107). SKP2 is a component of Skp1/Cullin/F-box protein ubiquitin ligase, which functions to degrade p27 (kip1) and inactivates cyclin-dependent kinases (Cdks). By interfering with TCR signaling pathway, PD-1 engagement also leads to changes in transcriptional program of T cells, as seen in the gene expression profiles from HIV-specific CD8+ T cells in HIV-infected individuals. Among the list of the regulated genes include basic leucine transcription factor, ATF-like (BATF), an AP-1 family of transcription factor, of which overexpression impairs T-cell proliferation and cytokine functions. In contrast, BATF silencing rescue HIV-specific T cells derived from individuals with chronic viremia (80).

## Epigenetic alternations of T-cell exhaustion

Several reports have described that the epigenetic landscape of a cell directly influences the transcriptional regulation of T-cell exhaustion. Therefore, decoding the language of epigenome specific to exhausted T cells appears to be one of the fundamental steps toward developing therapeutic strategies for overcoming T-cell exhaustion (108).

Currently, there is a paucity for information on global epigenetic landscape for exhausted T cells although recent studies of the *Pdcd1* locus (which encodes PD-1) revealed important information (29). During acute LCMV infection, the regulatory region of the *Pdcd1* locus is completely demethylated in exhausted CD8+ T cells compared to effector and memory T cells counterparts, and that reduction of virus titers is unlikely to affect methylation pattern as the *Pdcd1* regulatory region remained unmethylated when virus titers decreased (109). Moreover, chromatin remodeling of CD8+ T cells from LCMV-infected mice revealed that diacetylated histone H3 was downregulated in total and virus-specific CD8+ T cells suggesting loss of epigenetically active genes (110). Intriguingly, *in vitro* treatment of exhausted CD8+ T cells with histone deacetylase (HDAC) inhibitors improved function of exhausted T cells in this recent study.

Effector T cells and memory T cells display a number of chromatin accessible regions (ChARs) within the *Ifng* locus which are absent in exhausted T cells. However, ChARs of the exhausted T cells are only moderately altered after treatment with PD-L1 inhibitor (111). Assessment of the epigenetic state during T-cell exhaustion in acute and chronic infections has identified a number of notable changes to the chromatin accessibility unique to exhausted T cells, which were marked by distinct regulatory sequences possessing characteristics of enhancer, such as enrichment at the intergenic and intronic regions, depletion of transcription start sites (TSS), as well as gene-distal regulatory elements (21, 111). These ChARs were grouped into modules, which upregulate adjacent genes, including *Pdcd1*. Of note, deletion of an −23.8 kb ChAR harboring transcription factors binding motifs of Sox3, Tbx21 (Encodes T-bet), and Rara (encodes for retinoic acid receptor, RAR) led to dramatic reduction in the expression of PD-1, illustrating its role as an enhancer in PD-1 regulation (21). These described observations, along with other studies (112, 113) indicate that continued antigen burden imparts a stable pattern of chromatin accessibility in exhausted T cells with functional consequences on transcription factors. Nevertheless, the molecular basis by which transcription factors exert their influence on cell fate remains unclear (3).

Exhausted CD8+ T cells reinvigorated by PD-L1 blockade have a distinct epigenetic profile in comparison with memory T cells (111). This epigenetic state is reported to be maintained after PD-1/PD-L1 blockade. Subsequent work involving transferring exhausted T cells from chronically infected mice with LCMV to mice with resolved acute infection showed sustained PD-1 expression as well demethylation of PD-1 promoter in the exhausted CD8+ T cells post transfer (114). Youngblood and colleagues investigated the capacity of HIV-specific CD8+ T cells to modify the PD-1 epigenetic program after reduction in viral load. They reported that PD-1 promotor region was unmethylated in the PD-1 ^hi^ virus-specific CD8+ T cells, whereas it remained methylated in donor-matched naive cells at acute and chronic stages of HIV infection. Interestingly, the transcriptional regulatory region sustained unmethylated in virus-specific T cells from individual with a viral load controlled by ART or from elite controllers (115).

## Metabolic programming in T-cell exhaustion

An increasing body of evidence indicates that sufficient nutrient supply and energy generation are main determinants of the T cell's ability to proliferate and mediate effector function (116, 117). Although alternations in the transcription program of T cells be linked to T-cell exhaustion, several reports have also suggested that metabolic deficiency and deregulation of nutrient sensing signaling pathways contribute to T-cell exhaustion (12, 118, 119).

Bengsch et al. have recently demonstrated that glycolytic and mitochondrial metabolism in early effector CD8+ T cells is inhibited by PD-1 signaling in chronic LCMV infection. They reported that PD-1 signals inhibit the expression of key metabolic regulator peroxisome proliferator-activated receptor gamma coactivator 1-alpha (PGC1α) and interestingly overexpression of PGC1α corrected some metabolic alterations in developing exhausted T cells and improved effector function (120). Importantly, the authors of this study also reported that T-bet^Hi^PD-1^Int^ cells have higher glucose uptake and a decrease in mitochondrial mass in comparison to Eomes^Hi^PD-1^Hi^ cells and only PD-1^Int^ cells could be rescued metabolically and functionally.

Naive and resting T cells make use of fatty acid oxidation (FAO) and the mitochondrial tricarboxylic acid (TCA) cycle to generate large amounts of ATP through oxidative phosphorylation (OXPHOS) (121, 122). Recent studies in a murine model revealed that mitochondrial activity was one of the requirements for the activation and sustenance of antigen-specific responses (123, 124). Upon activation, T cells switch their metabolism to high rates of glycolysis even in the presence of sufficient oxygen and this support proliferation and effector function via providing fast energy and metabolites (125).

HIV-specific T cells upregulated OXPHOS owing to an increased mitochondrial mass (126). However, it's not clear whether the observed increase was due to an augmented number of functional mitochondria or the emergence of massive non-functional mitochondria (127, 128). Schurich et al. recently described that the poorly functional PD-1^hi^ T cell responses against HBV upregulate GLUT1, which is a constitutive glucose transporter (116). They also showed that Glut1^hi^ HBV-specific T cells are dependent on glucose supplies, unlike the more functional CMV-specific T cells that could utilize OXPHOS in the absence of glucose. The exhausted HBV-specific T cells were unable to switch to OXPHOS and had also increased mitochondrial size and lower mitochondrial potential, all suggestive of mitochondrial dysfunction (116). Intriguingly, their defect in mitochondrial metabolism was rescued by the proinflammatory cytokine interleukin (IL)-12, which recovered the exhausted HBV-specific T cell effector function, increased their mitochondrial potential, and reduced their dependence on glycolysis.

**Figure 2:**
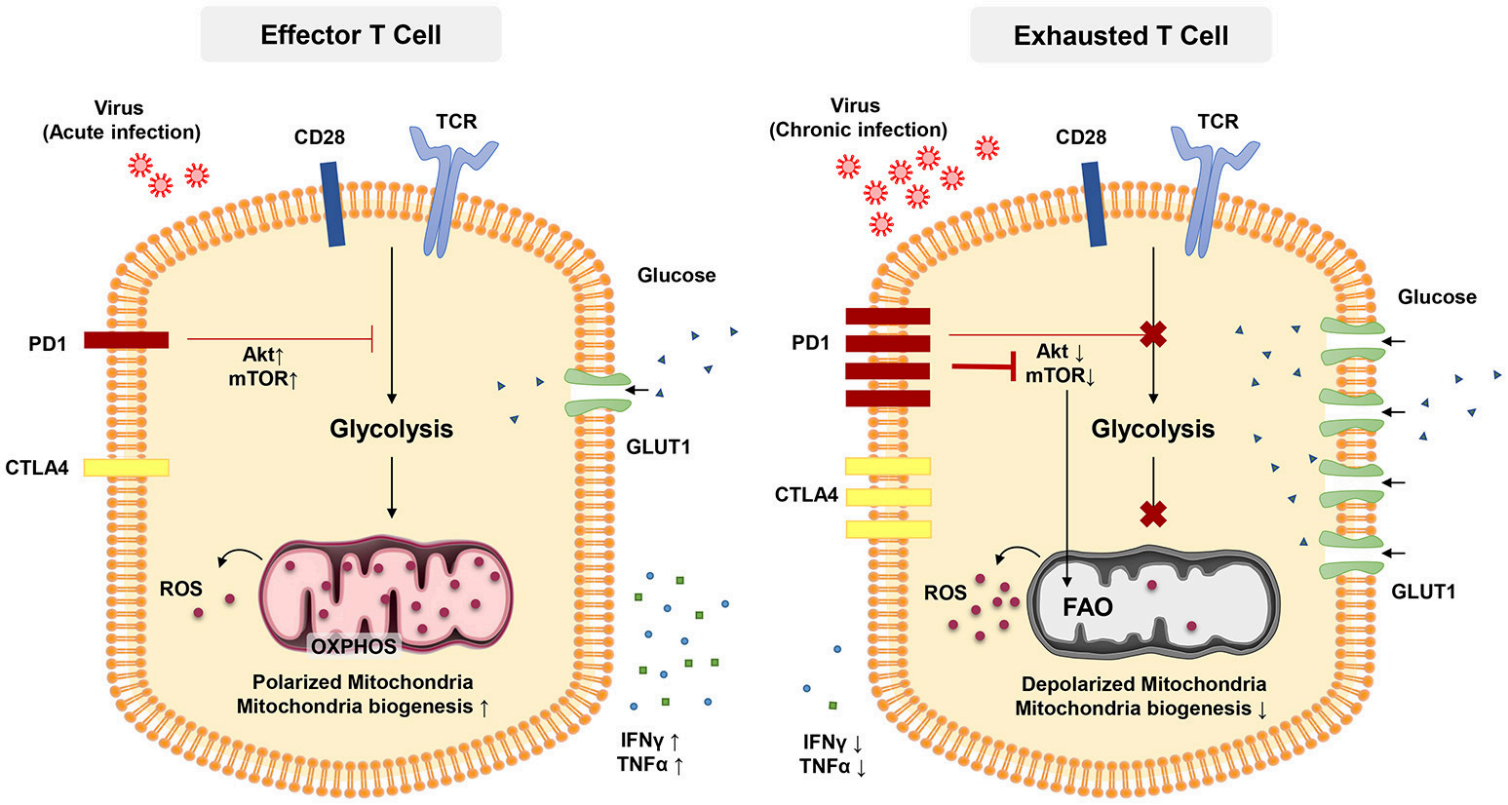
In acute infection, effector CD8 + T cells induce glycolysis after activation to sustain effector functions. Akt and mTOR promote glycolysis and support effector T cell functions. In chronic infection, exhausted T cells express inhibitory receptors such as PD-1 and CTL4. PD-1 signaling reduces AKT activation and thus suppress mTOR activity, switching T cell metabolism from glycolysis to FAO. These metabolic reprogramming may lead to mitochondrial depolarization, reduction of mitochondrial biogenesis and higher rate of ROS production which is associated with functional impairment in exhausted T cells.

The transcription factor mTOR is a key molecule sensing ATP and intracellular amino acids (129) that also regulates the fatty acid metabolism in memory T cells (122). The PD-1 signaling pathway also affects T-cell functions through metabolism. PD-1 signaling reduces AKT activation and thus suppress mTOR activity, switching T cell metabolism from glycolysis to FOA (Figure 2) (84, 130, 131). Declined mTOR activation in exhausted PD-1+ CD8+ T cells give rise to increased activity of the transcription factor forkhead box O1 (FoxO1), which sustains PD-1 expression and survival of exhausted CD8 T cells (84). A recent report indicated that IL-12 enhanced mTOR expression in antigen-stimulated CD8+ T cells, thereby modulating CD8+ T effector differentiation and metabolism. Furthermore, blockade of the mTOR signaling pathway via rapamycin inhibits IL-12-induced expression of T-bet and skews the CD8+ T cell response toward EOMES dependent memory development (132). Notably, T-bet has a significant role in sustaining the limited effector functional capacity of T cells in chronic infections (81). Moreover, T-bet expression is increased by IL-12 in exhausted T cells in chronic HBV infection and this enhance their functionality (133).

Targeting metabolic dysfunctions with mitochondrion-targeted antioxidants are reported to remarkably improve the antiviral activity of exhausted HBV-specific CD8+ T cells suggesting the pivotal role for ROS in T-cell exhaustion (134). It is crucial to further investigate the contributions of mitochondrial biogenesis on T-cell exhaustion and how we can target mitochondrial metabolism of T cells when treating chronic viral infection (135).

## Other potential strategies to overcome T-cell exhaustion

Engineered T cells, such as T cells expressing chimeric antigen receptors (CARs), are another strategy to overcome exhaustion in cancer and chronic infections (136–138). Nevertheless, CAR-T cells also become exhausted and require immune checkpoint blockade so that they can restore their functionality (139, 140). Instead of targeting the PD-1/PD-L1 signaling pathway, PD-1 expression could be declined by gene editing approach, made possible by the CRISPR-Cas9 system. A recent report showed a decreased PD-1 expression on primary human cells, utilizing the CRISPR-Cas9 system (141), demonstrating a method to generate CAR-T cells with more resistance to exhaustion. Nonetheless, as up-regulation of inhibitory receptors represents a way for the immune system to restrict immunopathology triggered by prolonged exposure to antigen, a more fine-tuned and adjustable approach to avoid exhaustion could be preferable (142–150).

Recently a stem cell–like CD8+ T-cell subset was discovered among exhausted PD-1-expressing CD8+ T cells during chronic viral infection and this subset can expand in response to PD-1-targeted immunotherapy in contrast with terminally differentiated/exhausted PD-1-expressing CD8+ T cells (151). These two CD8+ T cell populations have distinct expression profiles of co-inhibitory receptors and co-stimulatory molecules, so describing how different immunotherapeutic interventions affect these two population is highly relevant for understanding the mechanistic basis of the efficacy of present and future immunotherapies that target exhausted T cells (29).

## Conclusions

Our understanding of T-cell exhaustion is advancing at a rapid pace. However, it remains unclear as to what key transcription factors are involved in critical signaling pathways related to exhaustion and how these transcription factors are regulated by epigenomic alterations. Moreover, most of the studies of T-cell exhaustion have been focused on LCMV model and the critical changes in T cell phenotype and functional impairment of exhausted T cells utilizing human infected samples has been neglected due to lack of *in vitro* models. Finally, more detailed understanding of human anti-viral immunity is still critical to develop novel immunotherapies to reverse the state of exhaustion.

## Author contributions

All authors listed have made a substantial, direct and intellectual contribution to the work, and approved it for publication.

### Conflict of interest statement

The authors declare that the research was conducted in the absence of any commercial or financial relationships that could be construed as a potential conflict of interest.
